# Characteristics and management of the offending veins in microvascular decompression surgery for trigeminal neuralgia

**DOI:** 10.1007/s10143-020-01411-2

**Published:** 2020-10-27

**Authors:** Hidetoshi Kasuya, Shigeru Tani, Yuichi Kubota, Suguru Yokosako, Hidenori Ohbuchi, Naoyuki Arai, Mayuko Inazuka, Mikhail Chernov

**Affiliations:** Department of Neurosurgery, Medical Center East, Tokyo Women’s Medical University, 2-1-10 Nishiogu, Arakawa-ku, Tokyo, 116-8567 Japan

**Keywords:** Microvascular decompression, Offending veins, Outcome, Root entry zone, Surgical technique, Trigeminal neuralgia

## Abstract

The optimal technique of microvascular decompression (MVD) for trigeminal neuralgia (TN) caused by venous conflict remains unclear. The objectives of this study are to characterize the offending veins identified during MVD for TN and to evaluate intraoperative technique applied for their management. From 2007 till 2019, 308 MVD surgeries were performed in 288 consecutive patients with TN, and in 58 of them, pure venous conflict was identified. In 44 patients, the offending vein was interrupted, as was done for small veins arising from the cisternal trigeminal nerve (CN V) or its root entry zone (REZ) causing their stretching (19 cases), small veins on the surface of REZ (9 cases), transverse pontine vein (TPV) compressing REZ or distal CN V (12 cases), and superior petrosal vein (SPV) using flow conversion technique (4 cases). In 14 other cases, the offending vein was relocated, as was done for the SPV or the vein of cerebellopontine fissure (8 cases), TPV (3 cases), and the vein of middle cerebellar peduncle (3 cases). Complete pain relief after surgery was noted in 49 patients (84%). No one patient experienced major neurological deterioration. Postoperative facial numbness developed in 14 patients (24%), and in 8 of them, it was permanent. In 14 patients, MRI demonstrated venous infarction of the middle cerebellar peduncle, which was associated with the presence of any (*P* = 0.0180) and permanent (*P* = 0.0002) facial numbness. Ten patients experienced pain recurrence. Thus, 39 patients (67%) sustained complete pain relief at the last follow-up (median, 48 months), which was significantly associated with the presence of any (*P* = 0.0228) and permanent (*P* = 0.0427) postoperative facial numbness. In conclusion, in cases of TN, small offending veins arising from REZ and/or distal CN V and causing their stretching may be coagulated and cut. In many cases, TPV can be also interrupted safely or considered as collateral way for blood outflow. The main complication of such procedures is facial numbness, which is associated with the venous infarction of middle cerebellar peduncle and long-term complete pain relief.

## Introduction

Compression of the cisternal segment of trigeminal nerve (CN V) by the adjacent veins is widely recognized as one of the possible causes of trigeminal neuralgia (TN). However, only few studies highlighted the details of microvascular decompression (MVD) surgery in such cases [3, 5–8, 16, 22–24]. Although venous conflict may differ from more common arterial compression, the characteristics of offending veins affecting CN V, its root entry zone (REZ), or porus trigeminus have not been described thoroughly. Similarly, the optimal surgical technique for such cases remains unclear, and no standard guiding principles for the intraoperative management of offending veins causing TN have been established to date. While some surgeons are certain with cutting all veins obscuring the access to and/or offending CN V, others put all efforts to preserve these vessels for avoidance of possible complications caused by the interruption of blood outflow [4, 7, 17, 18, 21]

The program of MVD surgeries for TN has been established in the Department of Neurosurgery, Medical Center East, Tokyo Women’s Medical University more than 10 years ago and presumes consistent collection of all relevant clinical, radiological, surgical, and follow-up information in the prospectively maintained computer database. From the most beginning of this initiative, the particular interest was put on cases with pure venous conflict. Our experience with characterization of veins offending CN V in patients operated on for TN and evaluation of the surgical technique applied for the management of these vessels with regard to both short- and long-term postoperative outcomes are presented herein.

## Materials and methods

From April 2007 till December 2019, 308 MVD surgeries were performed in 288 consecutive patients with TN admitted to our center. During 19 primary or repeat procedures, no neurovascular conflict was identified intraoperatively; in 111 cases, the compressing artery was noted; in 120 cases, the neurovascular conflict was predominantly caused by the compressing artery, but offending veins were presented as well; and in 58 surgeries performed in 58 patients, only offending vein(s) were identified. The latter cases have formed the study cohort of the present retrospective analysis. All evaluated data were extracted from the prospectively maintained computer database, as well as from the surgical records and intraoperative videos. Research protocol was approved by the Institutional Review Board of Tokyo Women’s Medical University (No. 2741).

### Clinical data

The study cohort comprised 39 women (67%) and 19 men (33%) aged from 17 to 81 years (mean age, 59.4 ± 15.7 years); 17 patients (29%) were younger than 50 years. The duration of symptoms varied from 6 months to 30 years (mean, 6.4 ± 7.0 years), and in 21 cases (36%), it was less than 3 years. Typical clinical presentation of TN with paroxysmal “electric discharge”-like facial pain with certain unilateral topographical distribution within the one or more divisions of CN V triggered by the facial stimulation was noted in 52 patients (90%). In other 6 patients (10%), the clinical symptoms were considered atypical (e.g., the pain was continuous or “burning”). Pain was located on the right and left side of the face in 36 (62%) and 22 (38%) patients, respectively. It was localized solely within the areas of ophthalmic (V1), maxillary (V2), and mandibular (V3) divisions of CN V in 2 (3%), 19 (33%), and 15 (26%) cases, respectively, whereas in 7 cases (12%), pain included areas of V1 and V2, in 14 (24%) of V2 and V3, and in 1 case (2%) of all three divisions (V1/V2/V3). The indications for surgical treatment included insufficient control of pain at the optimal dose and schedule of carbamazepine (Tegretol^®^) administration or associated side effects. In all cases, MRI excluded presence of the structural intracranial lesion or identifiable neurovascular conflict.

### Surgical procedure

Fifty patients (86%) in the study cohort underwent MVD procedure for newly diagnosed TN, whereas in 8 others (14%), surgery was done for pain recurrence. Informed consent was provided before intervention by each patient and his/her nearest family member.

The consistent goal of surgical treatment was the elimination of vascular compression or kinking of REZ and distal CN V. The insertion of the separating prostheses was avoided, and instead the offending vessel was relocated distally and fixed (usually to the tentorium or dura mater on the petrous bone) in new position by fibrin glue with or without use of the small piece of Teflon [11]. Although all attempts were constantly put on preservation of the superior petrosal vein (SPV) and its main tributaries, it was acknowledged that in some cases MVD could be hardly completed without coagulation and cutting of small veins. No intentional lesioning of REZ and distal CN V was done in any case.

All surgical procedures were performed under general anesthesia with the use of retrosigmoid approach and auditory brainstem response (ABR) monitoring. The patient was placed in a park-bench position. After straight incision of the soft tissues, craniectomy with diameter of 2.5–3 cm bordering transverse and sigmoid sinuses was created. T-shaped dural incision was made, and cerebrospinal fluid was withdrawn from the subarachnoid cisterns. The great horizontal fissure of the cerebellum was dissected (unless it was adhered too tightly), and superior semilunar lobule was gently retracted with the suction tube, which allowed for access to REZ and distal CN V. By wide opening of the arachnoid and dissection of its membranes, the main trunk of SPV and its tributaries were freed as much as was needed in each individual case. The entire length of CN V from the pons to the porus trigeminus was inspected for the presence of offending vessel(s), which was facilitated in some cases by drilling the suprameatal tubercle off. If arterial compression was revealed, it was managed appropriately. If arterial compression was not apparent, the thorough search for offending veins and their management were done.

### Postoperative follow-up

Head CT next day after surgery was performed routinely in all cases. MRI examination was done in 51 patients (88%), usually 1 month after surgery or earlier, if new neurological symptoms were evident. Subsequent follow-up was carried on in the outpatient clinic. In addition, in April 2020, a survey with standard questionnaire addressed via postal mail or phone calls was accomplished for the evaluation of long-term outcomes. Pain relief and facial numbness were assessed according to the proposal for standardized analysis of the results of MVD surgery for TN [13] and the Barrow Neurological Institute (BNI) Pain Intensity and Facial Numbness Scores [20]. Recurrence was defined as any degree of facial pain relapse after more or less prolonged pain-free period after surgery. The length of postoperative follow-up varied from 5 to 139 months (mean, 55 ± 38 months; median, 48 months).

### Statistics

Two-tailed Fisher exact test and ANOVA were used for data analysis. Proportion of patients with complete pain relief during follow-up was evaluated with the Kaplan-Meier method. The level of statistical significance was defined at *P* < 0.05. All calculations were performed with the commercially available software JMP^®^ Pro 14 (SAS Institute Inc.; Cary, NC).

## Results

In comparison with cases with the apparent compressing artery identified during MVD surgery for TN (Table 1), patients in the study cohort (i.e., with pure venous conflict) were significantly younger (*P* = 0.0206) and more frequently underwent surgery for pain recurrence (*P* = 0.0089). In addition, in all patients with atypical clinical symptoms, pure venous conflict was identified intraoperatively, and in neither such case, a compressing artery was revealed (*P* < 0.0001).

**Table 1:** Comparison of 3 subgroups of patients with different types of the neurovascular conflict identified during 289 MVD surgeries for trigeminal neuralgia performed between April 2007 and December 2019

Clinical characteristics	Type of the neurovascular conflict*			*P* value
	Compressing artery only (*N* = 111)	Predominant compressing artery with presence of offending veins (*N* = 120)	Offending veins only (*N* = 58)	
Mean age (years)	65.8	63.6	59.4	0.0206
Gender (women/men)	65/46	77/43	39/19	0.4882
Mean duration of symptoms (years)**	7.0	6.0	6.4	0.6060
Cases with atypical symptoms (*N*)	0	0	6	< 0.0001
Side of pain (right/left)	65/46	73/47	36/22	0.8916
Pain distribution (*N*)***				0.6695
V1	5	5	2	
V2	32	41	19	
V3	21	27	15	
V1/V2	13	17	7	
V2/V3	26	19	14	
V1/V2/V3	11	8	1	
Surgeries for pain recurrence (*N*)	2	8	8	0.0089

Overall, 11 patients were operated twice and 2 patients were operated bilaterally

*N* number of cases

*During additional 19 primary or repeat procedures, no neurovascular conflict was identified intraoperatively

**Information on 49 cases was missed

***Information on 6 cases was missed

### Characterization and management of the offending veins during surgery

**Fig. 1:**
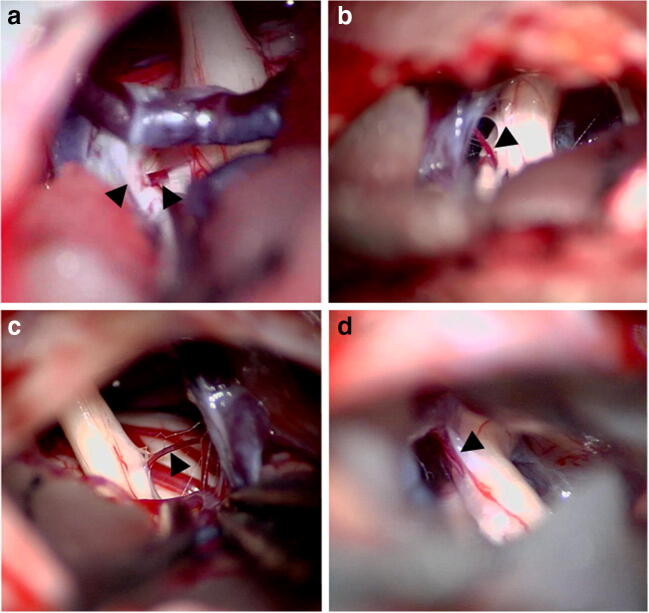
Intraoperative photographs during MVD procedures for trigeminal neuralgia demonstrate small veins (arrowheads) arising directly from the root entry zone (**a**) and the cisternal segment of trigeminal nerve (**b**–**d**) and stretching them. In all cases, the offending vessel was coagulated and cut, which resulted in complete pain relief immediately after surgery

**Fig. 2:**
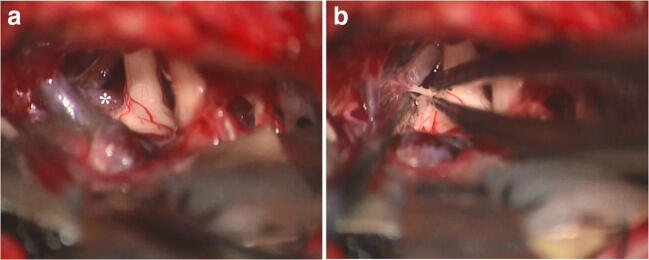
Intraoperative photographs during repeat MVD surgery in a 55-year-old woman with right-side trigeminal neuralgia. At the time of initial procedure, arterial neurovascular conflict caused by the vertebral artery has been released, and while offending vein was identified as well, its specific management was not done. Postoperatively, pain relief was achieved, but symptoms recurred 2 years later. During reoperation, stretching of the root entry zone of trigeminal nerve by the transverse pontine vein (**a**; asterisk) was revealed, and this vessel was coagulated (**b**) and cut. Complete pain relief was noted immediately after surgery, and no recurrence was marked during 10 years of subsequent follow-up

**Fig. 3:**
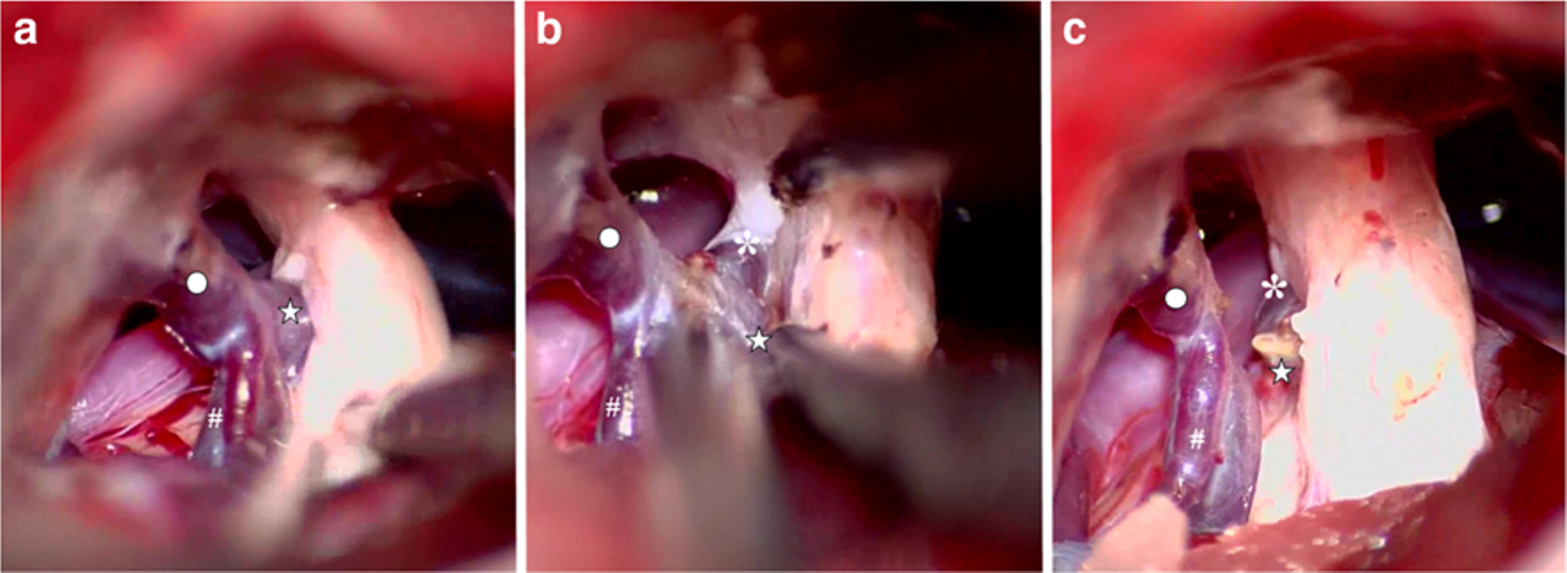
Intraoperative photographs during MVD surgery in a 67-year-old man with right-side trigeminal neuralgia before (**a**), during (**b**), and after (**c**) coagulation and cutting of the main trunk of vein of cerebellopontine fissure (VCPF; star), utilizing flow conversion technique allowed by the presence of collateral venous outflow. The VCPF penetrated through the cisternal segment of trigeminal nerve and converted into the superior petrosal vein (SPV; ring) together with the transverse pontine vein (asterisk), whereas there is also a tributary to SPV by the anterior lateral marginal vein (pound). Complete pain relief was noted immediately after surgery and was not accompanied by any complication

In 44 patients (76%), the offending vein was coagulated and cut (Figs. 1, 2, and 3), as was done for small veins arising directly from REZ or distal CN V and causing their stretching on the way toward SPV (19 cases), small veins on the surface of REZ (9 cases), transverse pontine vein (TPV) compressing REZ or distal CN V (12 cases), and SPV itself (4 cases) with the use of flow conversion technique, i.e., if sufficient blood outflow could be expected through collateral TPV.

In 14 other cases (24%), the offending vein was relocated and fixed in new position with fibrin glue, as was done for the main trunk of SPV or the vein of cerebellopontine fissure (VCPF) in 8 cases, TPV (3 cases), and the vein of middle cerebellar peduncle (3 cases). Nevertheless, even if these major veins have been transposed, their small tributaries arising from the pons, REZ, or distal CN V frequently should be coagulated and cut.

Outcomes after MVD surgeries for TN caused by pure venous conflict with regard to type of the offending vein management are summarized in Table 2.

**Table 2:** Outcomes after MVD surgery for trigeminal neuralgia caused by pure venous conflict with regard to type of the offending vein management

Management of the offending vein	Characteristics of the offending veins	Number of patients			Pain relief after surgery		Facial numbness after surgery		MRI signs of the venous infarction of middle cerebellar peduncle	Pain recurrence
		Overall	With atypical symptoms	Operated on for pain recurrence	Immediate	Delayed	Any	Permanent		
Coagulation and cutting (*N* = 44)	Small veins arising directly from REZ or distal CN V and causing their stretching	19	1	1	15	1	3	1	2	4
	Small veins on the surface of REZ	9	3	4	5	0	3	3	3	0
	TPV compressing REZ or distal CN V	12	1	2	10	0	2	1	2	4
	SPV (flow conversion technique)	4	0	0	4	0	1	1	2	0
Transposition and fixation (*N* = 14)	Main trunk of SPV or VCPF	8	1	1	7	1	3	2	4	1
	TPV	3	0	0	3	0	1	0	0	1
	VMCP	3	0	0	3	0	1	0	1	0
Overall	-	58 (100%)	6 (10%)	8 (14%)	47 (81%)	2 (3%)	14 (24%)	8 (14%)	14 (27%)*	10 (20%)**

*CN V* cisternal segment of the trigeminal nerve, *REZ* root entry zone, *TPV* transverse pontine vein, *SPV* superior petrosal vein, *VCPF* vein of the cerebellopontine fissure, *VMCP* vein of the middle cerebellar peduncle, *N* number of cases

*Calculated for 51 patients, in whom postoperative MRI was done

**Calculated for 49 patients with complete pain relief after surgery

### Postoperative pain relief

Complete pain relief (E-0 [13] or BNI Pain Intensity Score I [20]) was attained in 49 patients (84%), either immediately after surgery (47 cases) or later on (at 2 and 36 months; 2 cases).

### Surgical morbidity

No surgical complications, major neurological deterioration, or cerebellar hemorrhage or swelling after MVD procedure was noted in any patient of the study cohort.

Postoperative facial numbness was revealed in 14 patients (24%), and in 8 of them, it persisted at the time of the last follow-up examination or response to survey. In cases of permanent facial numbness, it was considered not bothersome (C-1 [13] or BNI Facial Numbness Score II [20]) and problematic for daily life (C-2 [13] or BNI Facial Numbness Score III or IV [20]) in 6 and 2 patients, respectively. The rate of any (*P* = 0.2907) or permanent (*P* = 1.000) facial numbness did not differ significantly between subgroups of patients in whom interruption or relocation of the offending vein was done.

**Fig. 4:**
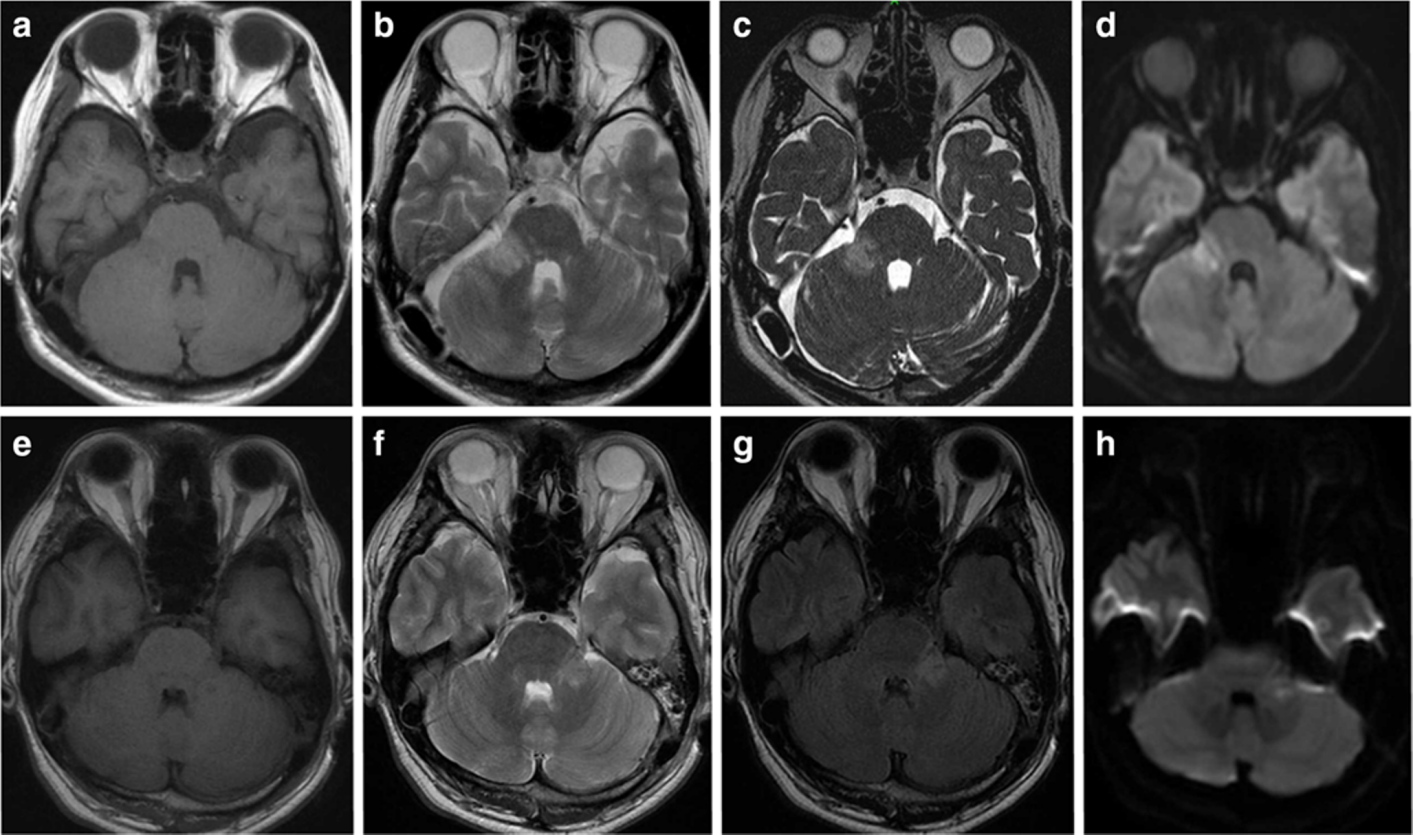
Illustrative cases of the venous infarction of middle cerebellar peduncle after MVD surgery. Upper row: a 47-year-old woman with the right-side trigeminal neuralgia (TN) and pain localization within the V3 area. Pain relief was noted postoperatively, but mild facial numbness has appeared within the same area. During surgery, superior petrosal vein (SPV) running across and compressing the root entry zone (REZ) was dissected, mobilized, relocated, and fixed to the dura on the petrous bone using small piece of Teflon and fibrin glue; however, small SPV tributary arising from REZ was coagulated and cut. T1-weighted (**a**), T2-weighted (**b**), constructive interference in steady state (**c**), and diffusion-weighted (**d**) images at 7 days after surgery demonstrate the venous infarction of right middle cerebellar peduncle. Facial numbness regressed completely within 1 month thereafter. Lower row: a 38-year-old man with left-side TN and pain localization within the V1/V2 area. Pain relief was noted postoperatively, but facial numbness has appeared within the left V2/V3 area. It was revealed during surgery that two SPV have compressed REZ from below, and one of these vessels was coagulated and cut, whereas the second one was transposed toward the porus trigeminus. T1-weighted (**e**), T2-weighted (**f**), fluid attenuation inversion recovery (**g**), and diffusion-weighted (**h**) images at 7 days after surgery demonstrate the venous infarction of left middle cerebellar peduncle. Facial numbness regressed completely within the V2 area, but persisted within the V3 area

In 7 patients with facial numbness, including 6 in whom this complication was permanent, postoperative MRI demonstrated area of mild-to-moderate hyperintensity on T2-weighted, constructive interference in steady state (CISS), fluid attenuation inversion recovery (FLAIR), and diffusion-weighted (DWI) images within the middle cerebellar peduncle, considered as the venous infarction (Fig. 4). There was statistically significant association of this imaging finding with any (*P* = 0.0180) and permanent (*P* = 0.0002) postoperative facial numbness (Table 3 presents data for 51 patients in whom postoperative MRI was done). Additionally, in 7 other cases, such imaging finding was not accompanied by any symptoms. According to routine neurological examination, patients with the venous infarction of middle cerebellar peduncle neither had specific complaints on, nor any objective sign of the cerebellar dysfunction. The incidence of the venous infarction of middle cerebellar peduncle revealed by postoperative MRI, either symptomatic or asymptomatic, did not differ significantly between subgroups of patients in whom interruption or relocation of the offending vein was done (*P* = 0.2907).

**Table 3:** Association between facial numbness after MVD surgery for trigeminal neuralgia caused by pure venous conflict and signs of venous infarction of the middle cerebellar peduncle on postoperative MRI

Postoperative facial numbness	MRI signs of venous infarction of the middle cerebellar peduncle		
	Present (*N* = 14)	Absent (*N* = 37)	*P*-value
Transient and permanent numbness			0.0180
Yes (*N* = 12)	7 (14%)	5 (10%)	
No (*N* = 39)	7 (14%)	32 (63%)	
Permanent numbness only			0.0002
Yes (*N* = 6)	6 (12%)	0 (0%)	
No (*N* = 45)	8 (16%)	37 (73%)	

Calculated for 51 patients, in whom postoperative MRI was done

*N* number of cases

### Durability of pain relief

**Fig. 5:**
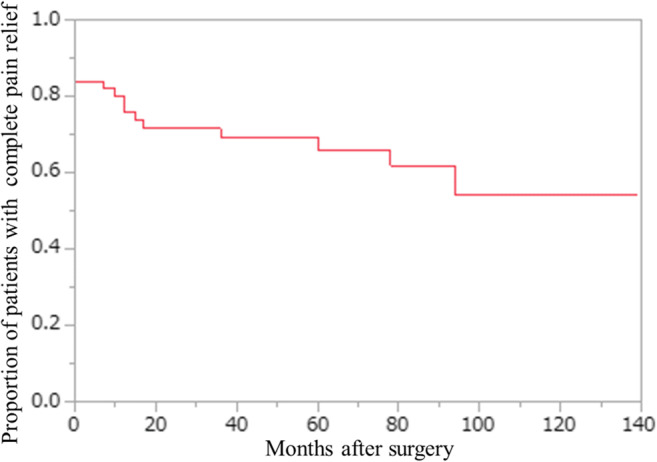
The durability of complete pain relief after MVD surgery for trigeminal neuralgia caused by pure venous conflict. Of note, this Kaplan-Meier curve was constructed without consideration of 2 patients demonstrating delayed (at 2 and 36 months) complete pain relief postoperatively

In 10 patients with complete pain relief after surgery, its recurrence was noted during follow-up (Fig. 5). Thus, 39 patients (67%) sustained complete pain relief (E-0 [13] or BNI Pain Intensity Score I [20]) at the time of last follow-up. Long-term pain-free status showed statistically significant association with the presence of any (*P* = 0.0228) and permanent (*P* = 0.0427) postoperative facial numbness (Table 4).

**Table 4:** Factors associated with sustained pain relief after MVD surgery for trigeminal neuralgia caused by pure venous conflict

Evaluated factors	Complete pain relief at the last follow-up (*N*)		*P*-value
	Yes	No	
Female gender (*N* = 39)	26	13	1.000
Age less than 50 years (*N* = 17)	14	3	0.1370
Duration of symptoms less than 3 years (*N* = 21)	15	6	0.7726
Atypical clinical symptoms (*N* = 6)	2	4	0.0828
Left side of facial pain (*N* = 22)	13	9	0.3899
Involvement of the V1 area into pain distribution (*N* = 10)	8	2	0.4720
Surgery for pain recurrence (*N* = 8)	5	3	0.5244
Any postoperative facial numbness (*N* = 14)	13	1	0.0228
Permanent postoperative facial numbness (*N* = 8)	8	0	0.0427
Total	39	19	-

*N* number of cases, *V1* the ophthalmic division of trigeminal nerve

### Management of pain recurrence

Overall, 5 of 10 patients with recurrent pain underwent repeat MVD procedure. During reoperation, new offending veins and/or small arteries were identified in all cases and were coagulated and cut, which resulted in complete pain relief in 4 patients. Among 5 patients who did not undergo surgery for recurrent pain, it was well tolerable with or without medication (E-1 or E-2 [13] or BNI Pain Intensity Score II or III [20]) in 2, but was not controllable (E-3 [13] or BNI Pain Intensity Score IV or V [20]) in 3.

## Discussion

It has been recognized that in 4–19% of cases, TN is caused by veins offending CN V [3, 5–8, 16, 21–24], whereas in our practice, pure venous conflict was identified in 58 of 289 consecutive MVD surgeries (20%) performed over a rather long-time span of > 12 years. A somewhat higher rate of this finding in comparison with previous studies may be related to the special interest of our group in the surgical management of such pathological condition and the thorough completing prospectively maintained computer database. Our patients with pure venous conflict were significantly younger than those in whom compressing artery was revealed, and two-thirds of them were women, which well corresponds to reports of others [1, 15]. In particular, in their recent analysis Magown et al. [15] also found that absence of identifiable neurovascular conflict on MRI in cases of typical TN is predominantly noted in younger women. Arterial compression of CN V is considered as the main cause of TN, and it has been suggested that development of atherosclerosis and vessel elongation during aging result in its shift toward the nerve [1]. However, venous conflict as etiological cause of TN may be more related to anatomical and congenital factors, thus frequently encountered in younger individuals [19]. In addition, the patients in the analyzed cohort significantly more often showed atypical clinical symptoms and more frequently underwent intervention for pain recurrence, which reflects difficulty of MVD surgery for TN in cases without evident arterial compression.

### Stretching veins as a cause of trigeminal neuralgia

Higher rate of pure venous conflict revealed in our series may be also related to the constant consideration of small veins not compressing, but stretching REZ or distal CN V. Such cause of TN has been overlooked by others, while it was noted in one-third of patients in the analyzed cohort and may be considered as one of the major findings of the present study.

Based on their experience with surgical management of TN without vascular compression, Ishikawa et al. [9] suggested that when the cisternal segment of CN V is fixed to the surrounding structures, the stretching force would be maximal at REZ, which may promote hyperexcitability. Similar pathophysiological mechanism may be considered in cases of stretching veins, presuming the necessity of nerve release for the achievement of pain relief. To the best of our knowledge, this is the first report describing the possibility of such cause of TN and suggesting its surgical correction.

### Transposition of the offending veins during MVD surgery

Surgical options in patients with TN without clear identification of the compressing artery on MRI are usually considered limited [15], but our experience demonstrates that in such cases, MVD may be indicated. Obviously, neurovascular conflict may be absent even in the presence of typical symptoms [9, 15], but rather often the intraoperative exploration may reveal offending veins. Although under such conditions, the success rate of intervention is generally lower than with arterial compression [21], application of appropriate surgical technique frequently results in complete pain relief.

The neurovascular conflict causing TN typically affects REZ at the origin of CN V from the pons, but in some cases, it may be located more distally. Therefore, meticulous inspection and dissection of the entire cisternal segment of CN V up to the porus trigeminus is absolutely necessary [17, 21]. In our practice, insertion of the separating prostheses is avoided, and instead the offending vessel is shifted away and fixed in new position to the dura mater by fibrin glue [11]. Such technique, standard in cases of compressing arteries, has been similarly applied for large offending veins, such as TPV, SPV, and their main tributaries. It was done in 14 patients (24%) of the present series and resulted in complete postoperative pain relief in all of them. However, even if the effective release of neurovascular conflict can be achieved by the relocation of large offending vein, still the smaller vessels frequently should be coagulated and cut. It may explain relatively high frequency (5 cases) of the venous infarction of middle cerebellar peduncle revealed on MRI after surgery in this subgroup.

### Safety of the offending veins interruption

In many cases, anatomical interrelationships between the offending vein and CN V do not allow for transposition of the vessel, and without its interruption release of the neurovascular conflict cannot be achieved [18]. Coagulation and cutting of the small offending veins during MVD surgery is generally considered sufficiently safe, and in the present series, it was done routinely for management of tiny vessels located on the surface of REZ or causing either stretching or kinking of REZ and distal CN V arising directly from them. The diameter of these vessels is much smaller than recommended thresholds (ranging from 1.3 to 2 mm [17]) for safe division of veins during cerebellopontine angle (CPA) surgery. However, the interruption of larger veins, such as TPV or SPV, may raise obvious concerns because of their presumed importance for blood outflow [17]. In particular, the reported rates of complications after cutting of SPV, the major bridging vein within the CPA, vary widely (from < 0.5 to 31%) and may manifest with the variety of symptoms ranging from mild to life-threatening [17, 18].

TPV has been described as a possible cause of nerve compression in the vicinity to porus trigeminus [3, 5, 7, 8, 16, 21], but was not noted previously as a reason for more proximal venous conflict. Anatomically, TPV is connected to the terminal vein and the anterior pontomesencephalic venous system, which is affiliated with the basal vein superiorly, the anterior medullary vein inferiorly, and the contralateral TPV, and this wide anastomosing network allows for sufficient collateral blood outflow [12]. In our opinion, coagulation and cutting of the interconnection between the anterior lateral marginal vein/SPV and the VCPF/TPV groups are sufficiently safe and may be rather effective as has been confirmed by results of the present study. Therefore, if sufficient collateral blood outflow through the adjacent veins is anticipated, such flow conversion technique should be considered and applied when it is deemed necessary for the achievement of complete MVD in patients with TN.

### Clinical outcomes

Pure venous conflict has been recognized as unfavorable prognostic factor for the surgical outcomes after MVD for TN and its association with the failure to attain complete pain relief was emphasized [1, 21]. Indeed, only 81% of our patients had complete pain relief immediately after surgery, and the rate of pain recurrence was not negligible (10 out of 49 cases with complete postoperative pain relief). Overall, only 39 patients (67%) who underwent treatment for pure venous conflict have sustained complete pain relief at the time of last follow-up. Although much lower in comparison with the similar rate after elimination of the arterial compression, it still seems sufficient enough to justify surgery in patients with severe TN resistant to medical therapy and absence of the neurovascular conflict on MRI.

Overall, 24% of the analyzed patients have experienced postoperative facial numbness, and in half of them it was permanent. In general, this complication is considered more common after MVD surgery for pure venous conflict [2, 8, 16]. Jawahar et al. [10] noted that most patients with postoperative facial numbness demonstrate changes in the cisternal segment of CN V or pons on MRI. It well corresponds to the findings of the present study. In 7 patients with facial numbness, including 6 in whom it was persisted at the time of last follow-up examination or response to survey, postoperative MRI demonstrated the venous infarction of middle cerebellar peduncle, and an association between these clinical and imaging findings was statistically significant. In 7 other cases, the venous infarction of middle cerebellar peduncle revealed on MRI was asymptomatic. Of note, incidence of the facial numbness and the venous infarction of middle cerebellar peduncle after surgery did not differ significantly between subgroups of patients in whom interruption or relocation of the offending vein was done, which may reflect the frequent necessity to coagulate and cut the smaller veins during transposition of the larger one.

To the best of our knowledge, no systematic analysis of the venous cerebellar infarctions after MVD surgery for TN caused by venous conflict has been reported previously. In the presented series, they were evaluated routinely with advanced MRI (including CISS, FLAIR, and DWI sequences), which was performed within the first month after surgery in 88% of cases. Overall frequency of the venous infarction of middle cerebellar peduncle was prominent, as corresponding imaging findings were marked in 27% of patients in whom examination was done. Nevertheless, while relatively high probability of this complication should be taken into consideration during planning of surgery and getting an informed consent from the patient, it should be noted that in neither case of our series the venous infarction of middle cerebellar peduncle has resulted in major neurological deterioration, nor cerebellar hemorrhage or swelling were observed. The incidence and clinical course of the venous cerebellar infarctions after MVD surgery for TN certainly require additional analysis in large-scale clinical investigations.

Notably, the present study identified a statistically significant association between postoperative facial numbness and long-term pain-free status. Out of 39 patients who sustained complete pain relief, 13 experienced postoperative facial numbness, and in 8 of them, it was permanent. In contrast, among 19 individuals who did not show complete pain relief after surgery or experienced pain recurrence, transient postoperative facial numbness was noted only in one. The association between development of facial numbness and pain relief has been frequently noted after destructive treatment of TN with stereotactic radiosurgery, glycerol or radiofrequency rhizotomy, and percutaneous balloon compression. However, it was not reported after MVD surgery and may represent a specific feature of such procedures directed at the release of pure venous conflict. In such cases, the interruption of small offending veins may result not only in release of the neurovascular conflict and elimination of the nerve stretching or kinking but also in inadvertent lesioning of the REZ and/or distal CN V, which may have an additional favorable impact on the outcome. Moreover, considering statistically significant associations of the postoperative facial numbness both with the venous infarction of middle cerebellar peduncle and with the durable complete pain relief demonstrated in our patients, it may be suggested that such treatment effect has been caused not only by the peripheral decompression itself, but in some part resulted from the central denervation in the brainstem, e.g., due to lesion within the principal sensory nucleus of CN V. The possibility of such pathophysiological mechanism should be taken into consideration and investigated further.

Finally, similar to Lee et al. [14], we have found that pain recurrence after more or less prolonged pain-free period after surgery was mainly caused by newly developed offending veins and/or small arteries. Importantly, their coagulation and cutting during repeat MVD has resulted in complete pain relief in 4 of 5 reoperated patients of the present series.

### Study limitations

The main limitations of the present study are related to its retrospective design, while extraction of all relevant information from the rigorously completed prospectively maintained computer database has significantly reduced the risk of relevant biases. The number of patients was relatively small; thus, statistical analysis may be somewhat underpowered. Finally, postoperative follow-up in some cases was short, and not all patients have responded to survey directed at the evaluation of long-term outcome, but it is most likely that the majority of those individuals who denied postoperative examinations and did not respond to questionnaire had sufficient pain relief after treatment.

## Conclusion

Surgical management of TN caused by pure venous conflict is challenging. According to our experience, small veins, which converge into SPV group, frequently arise directly from REZ and/or distal CN V and cause their stretching or kinking. These tiny offending vessels may be coagulated and cut. In many cases, TPV, and even SPV, can be also interrupted safely, while it should be done with certain caution and consideration of the individual details of vascular anatomy allowing for adequate collateral venous pathway if flow conversion technique is applied. Utilization of such intraoperative strategies in the present series has resulted in complete pain relief after surgery and on the long-term follow-up in 84% and 67% of patients, respectively. There was no one case of major complications or cerebellar hemorrhage or swelling. The venous infarction of middle cerebellar peduncle was revealed in 27% of patients, in whom postoperative MRI was done, and either manifested with facial numbness or was asymptomatic (half cases each). Temporary or permanent facial numbness after surgery was encountered rather often (24% of cases) and showed association with the venous infarction of middle cerebellar peduncle and the long-term complete pain relief.

## Data Availability

May be considered upon request.
